# Development and validation of a nomogram to predict anastomotic leakage in colorectal cancer based on CT body composition

**DOI:** 10.3389/fnut.2022.974903

**Published:** 2022-09-07

**Authors:** Shuai Xiang, Yong-Kang Yang, Tong-Yu Wang, Zhi-Tao Yang, Yun Lu, Shang-Long Liu

**Affiliations:** ^1^Department of Gastroenterology, Affiliated Hospital of Qingdao University, Qingdao, China; ^2^Department of Radiology, Affiliated Hospital of Qingdao University, Qingdao, China

**Keywords:** body composition, anastomotic leakage, colorectal cancer, prediction, nomogram

## Abstract

**Background:**

Anastomotic leakage (AL) is one of the most serious postoperative complications. This study aimed to investigate the predictive value of preoperative body composition for AL in patients with colorectal cancer (CRC).

**Methods:**

We first reviewed data from 3,681 patients who underwent radical CRC resection 2013–2021 in our hospital, and 60 patients were diagnosed with AL after surgery. We designed a nested case-control study and two controls were randomly selected for each case according to the time and position of surgery. Body composition was measured at the level of the third lumbar vertebra based on computed tomography (CT) images. The risk factors of AL were analyzed by univariate and multivariate analysis. Nomogram was built using binary regression analysis and assessed for clinical usefulness, calibration, and discrimination.

**Results:**

In the multivariate analysis, gender, blood glucose, nutrition risk screening (NRS), skeletal muscle area (SMA) and visceral fat area (VFA) were independent risk factors for developing anastomotic leakage after surgery. The prognostic model had an area under the receiver operating characteristic curve of 0.848 (95% CI, 0.781–0.914). The calibration curve showed good consistency between the predicted and observed outcomes. Decision curve analysis indicated that patients with colorectal cancer can benefit from the prediction model.

**Conclusions:**

The nomogram that combined with gender, blood glucose, NRS, SMA, and VFA had good predictive accuracy and reliability to AL. It may be conveniently for clinicians to predict AL preoperatively and be useful for guiding treatment decisions.

## Introduction

Colorectal cancer (CRC) is one of the most common cancers of the digestive system. According to statistics, CRC is the third most commonly diagnosed malignancy and the fourth leading cause of cancer-related deaths in the world (1). Currently, there are different types of treatment for patients with CRC, such as chemoradiotherapy, targeted therapy and surgery. However, according to SEER estimates for 2019, the 5-year survival rate for CRC in the United States was only 64.6% (2). High quality surgery is still the mainstay of CRC curative treatment.

Although the surgical techniques for CRC have improved significantly during the past decades, postoperative complications still plague surgeons. Among these complications, anastomotic leakage (AL) remains one of the most potentially life-threatening sequelae in CRC surgery due to its devastating impact on patients' mortality, short- and long-term morbidity, quality of life and survival, with the incidence ranging from 1% to 30% (3–6). Moreover, AL leads to the growth of health care costs and an extended hospital stay. It is well known that the etiology of AL is multifactorial. Previous studies have indicated that male sex, preoperative usage of steroids and elevated blood glucose were risk factors of AL (7, 8); however, all these factors lack specificity. Therefore, it is of great scientific and clinical significance to find and study more specific biomolecular markers for AL.

Computed tomography (CT) is a radiographic method commonly used in medical imaging. In terms of its usefulness in body composition measurement, it produces thin cross-sectional high-resolution images that can be processed to differentiate and measure volumes of fat and lean tissue. CT-based multiple body composition parameters have been used in various groups of patients to predict prognosis (9–12). Previous studies have showed that single-slice measurements at L2/L3 were strongly associated with total compartment volumes. Compared with body mass index (BMI), these parameters can fully reflect the detailed information of body composition and quantitatively reflect the density of skeletal muscle and adipose tissue.

Although almost all patients with CRC underwent abdominal CT examination prior to surgery, assessment of CT examination was limited to assessing tumor stage and the presence of distant metastases. The value of CT images reflecting body composition and patient fitness has not been used in clinical practice. Therefore, our purpose was to develop a helpful nomogram calculating each patient's individual probability based on predictive parameters of epidemiological, surgery-related data and laboratory parameters on the development of AL and examined the predictive value of body composition parameters.

## Materials and methods

### Study participants

A total of 3,681 patients received radical resection of CRC at our center between September 2013 and September 2021. There were 60 patients who were diagnosed with AL. We firstly performed a nested case-control study to identify the risk factors for AL, and two controls were randomly selected for each case according to the time (±1 month) and position (rectum or colon) of surgery. Patients who met the following criteria were included: (1) age ≥18 years; (2) primary colorectal adenocarcinoma confirmed by histopathology; (3) available abdominal CT examination within 2 wk before surgery; (4) no neoadjuvant chemoradiotherapy was performed before surgery. The exclusion criteria were as follows: (1) patients who had definite metastasis before surgery; (2) insufficient clinical information; (3) palliative resections. Finally, 180 patients with pathologically confirmed colorectal adenocarcinoma were included. All surgeries were performed with experienced gastrointestinal surgeons as the principal operator and in strict accordance with the standard surgical procedures. Smoking status was defined as smoking more than 1 cigarette a day for more than a year, regardless of their age at quitting and length of time since they quit. Alcohol status was defined as drinking more than twice a week for more than half of the year.

This retrospective non-intervention study was approved by the Ethics Committee of our hospital, and the requirement for informed consent was waived.

### Diagnosis of AL

AL was diagnosed based on clinical and radiologic manifestations: (1) suppurative or intestinal secretions through the drainage tube; or (2) the presence of air or abscess near the anastomotic site detected on CT; or (3) leakage found by X-ray contrast examination.

### Imaging analysis

**Figure 1:**
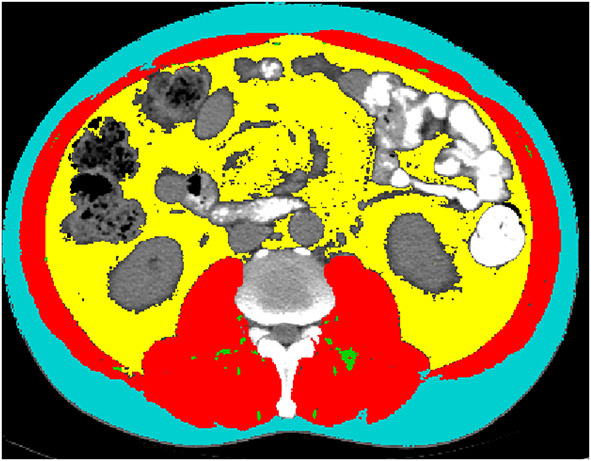
Example segmentations of subcutaneous fat area (SFA, turquoise), visceral fat area (VFA, yellow), intermuscular fat area (IMFA, green) and skeletal muscle area (SMA, red) at the third lumbar vertebra.

Preoperative CT images of all enrolled patients at the middle level of L3 were extracted from the institutional PACS (Picture Archiving and Communication System). All relevant images were anonymized, transferred to a personal computer, and analyzed using Tomovision's SliceOmatic (v5.0, Magog, Quebec, Canada). The CT HU thresholds were −29 to +150 for skeletal muscle area (13), −150 to −50 for visceral adipose and −190 to −30 for subcutaneous adipose tissue and intermuscular adipose (14, 15). Two experienced radiologists (T.Y.W and Z.T.Y) identified skeletal muscle and adipose tissue area independently. During the identification process, the radiologists were not aware of the occurrence of AL, which minimizes the bias. Intraclass correlation coefficient (ICC) was used to evaluate the interobserver measurement consistency for these parameters. These different body compositions are shown in Figure 1.

### Handling of missing data

Some data were missing for variables, including white blood cell (WBC), platelet, albumin (Alb), alanine aminotransferase (ALT) and aspartate aminotransferase (AST). We filled in missing data using the technique of multiple imputation by chained equations, which samples imputed values from the posterior predictive distributions of missing data. We assumed data were missing at random. The imputation model was specified on all predictors, outcomes, and dummy variables for study. Five imputations were carried out as this has a relatively high efficiency (16). We generated 5 datasets for analysis that were identical with respect to non-missing data but could vary on imputed values. In all, we imputed 38 of the 4,860 values (0.78%). All analyses were performed with the R software (version 4.1.2) using the rms and MICE packages.

### Statistical analysis

All statistical analyses were done by SPSS 25.0 software and R-software version 4.1.2. Pearson's chi-square test was used to analyze categorical variables. Student *t*-test or Mann-Whitney U test was used to analyze continuous variables according to normal distribution. Shapiro-Wilk test was used to check the normality. Univariate and multivariate analyses logistic regression analysis were used to determine independent risk factors. We carried out an internal verification process with 1,000 bootstrap resamples. The discrimination of the model was calculated using the area under the receiver operating characteristic curve (AUC), and the calibration power was analyzed using the calibration curve. Decision curve analysis (DCA) and clinical impact curves were used to calculate the net benefit. All tests were two-sided, and a value of *p* < 0.05 was considered to have statistical significance.

## Results

### Characteristics of included patients

A total of 180 patients [109 men and 71 women; age range, 31–89y; mean 62.80±11.419y (SD)] were included in the present study. All patients underwent radical resection of CRC through laparotomy or laparoscopy and 60 patients were diagnosed with AL among them. There were statistically significant differences in gender, WBC, Alb, blood glucose and NRS. The detailed characteristics of these patients are presented in Table 1.

**Table 1:** Clinical and histopathologic features of the patients.

**Patient characteristics**	**Non-AL**	**AL**	***Z***	***p***
**Surgical approach**, ***n*** **(%)**				0.268
Laparotomy	63 (52.5)	37 (61.7)		
Laparoscopy	57 (47.5)	23 (38.3)		
**Surgical site**, ***n*** **(%)**				1.000
Rectum	86 (71.7)	43 (71.7)		
Colon	34 (28.3)	17 (28.3)		
**Age, mean (SD), y**	62.94 (11.498)	62.17 (11.560)		0.671
**Gender**, ***n*** **(%)**				0.000^*^
Male	61 (50.8)	48 (80)		
Female	59 (49.2)	12 (20)		
**BMI, M (Q1~Q3), kg/m2**	24 (21.58~26.17)	25.1 (22.45~27.73)	−1.789	0.074
**T stage**, ***n*** **(%)**				0.398
T1	4 (3.3)	1 (1.7)		
T2	23 (19.2)	9 (15)		
T3	83 (69.2)	40 (66.7)		
T4	10 (8.3)	10 (16.7)		
**N stage**, ***n*** **(%)**				0.496
N0	74 (61.7)	32 (53.3)		
N1	31 (25.8)	17 (28.3)		
N2	15 (12.5)	11 (18.3)		
**Differentiation degree**, ***n*** **(%)**				0.248
Well	2 (1.7)	3 (5.0)		
Moderately	107 (89.1)	47 (78.3)		
Poor	11 (9.2)	10 (16.7)		
**Laboratory indicators**
**Neutrophil, M (Q1~Q3), 10** ^**9**^ **/L**	3.33 (2.68~4.44)	3.92 (2.91~5.07)	−1.788	0.074
**Lymphocyte, mean (SD), 10** ^**9**^ **/L**	1.68 (0.662)	1.65 (0.684)		0.794
**NLR, M (Q1~Q3)**	1.94 (1.42~3.13)	2.36 (1.64~3.49)	−1.849	0.065
**WBC, M (Q1~Q3), 10** ^**9**^ **/L**	5.87 (4.7~7.0)	6.61 (5.13~7.73)	−2.053	0.040^*^
**Platelet, M (Q1~Q3), 10** ^**9**^ **/L**	231 (203~281)	227.5 (194~293.75)	−0.005	0.996
**APTT, M (Q1~Q3), sec**	31 (28.2~34)	31.5 (28.95~34.47)	−0.857	0.391
**Alb, M (Q1~Q3), g/L**	40.6 (36.7~43.3)	38.49 (35.8~41.44)	−2.28	0.023^*^
**ALT, M (Q1~Q3), U/L**	12.5 (9.1~17)	14 (10.52~18.75)	−1.589	0.112
**AST, M (Q1~Q3), U/L**	14.1 (11.8~18.3)	15.65 (12.07~18.75)	−1.403	0.161
**AT-III, M (Q1~Q3), %**	95 (85~107)	90 (81.25~101.75)	−1.776	0.076
**Blood glucose, M (Q1~Q3), mmol/L**	4.9 (4.58~5.23)	6.71 (5.17~7.73)	−2.067	0.000^*^
**Hypertension**, ***n*** **(%)**				0.910
Yes	21 (17.5)	11 (18.3)		
No	99 (82.5)	49 (81.7)		
**CHD**, ***n*** **(%)**				0.837
Yes	7 (5.8)	4 (6.7)		
No	113 (94.2)	56 (93.3)		
**Smoking**, ***n*** **(%)**				0.320
Yes	38 (31.9)	24 (40)		
No	82 (68.1)	36 (60)		
**Drinking**, ***n*** **(%)**				0.381
Yes	31 (25.8)	20 (33.3)		
No	89 (74.2)	40 (66.7)		
**NRS**, ***n*** **(%)**				0.027^*^
<3	35 (29.2)	13 (21.7)		
≥3 and <5	78 (65.0)	36 (60.0)		
≥5	7 (5.8)	11 (18.3)		

AL, anastomotic leak; M (Q1~Q3), median (Q1~Q3); BMI, body mass index; NLR, neutrophil-lymphocyte ratio; WBC, white blood cell; PT, prothrombin time; APTT, activeated partial thromboplasting time; Alb, albumin; ALT, alanine aminotransferase; AST, aspartate aminotransferase; FIB, fibrinogen; AT-III, antithrombin III; CHD, coronary heart disease; NRS, nutrition risk screening.

* p < 0.05.

Median and Q1~Q3 of SMA, VFA, SFA, IMFA at the L3 spinal level, resulting from area-based quantification, were provided in Table 2. Among them, SMA and VFA showed statistical difference between AL and non-AL.

**Table 2:** Preoperative CT body composition of the patients.

	**Non-AL**	**AL**	***Z***	***p***
SMA, M (Q1~Q3), cm^2^	128 (111.6~153.7)	115.5 (99.04~129.42)	−3.230	0.001^*^
VFA, M (Q1~Q3), cm^2^	128.5 (84.28~174.8)	156.15 (117~202.77)	−2.845	0.004^*^
SFA, M (Q1~Q3), cm^2^	123.9 (83.83~175.5)	109.7 (87.41~139.15)	−1.014	0.310
IMFA, M (Q1~Q3), cm^2^	2.65 (1.49~4.37)	2.07 (1.07~3.63)	−1.630	0.103

SMA, skeletal muscle area; VFA, visceral fat area; SFA, subcutaneous fat area; IMFA, intermuscle fat area.

* p < 0.05.

#### Tests of the application presuppositions of the logistic model

According to the results of the univariate analysis in Tables 1, 2, seven factors with *p* < 0.05 were significantly related to AL, namely gender, WBC, Alb, blood glucose, NRS, SMA and VFA. Before incorporating multivariate regression analysis, we examined these factors for linearity test, multi-collinearity and influential point.

#### Linearity test

Box-Tidwell transformation was used for this test. A total of 12 factors were included in the analysis in this study, including 5 continuous variables and their respective natural logarithm (ln), and 2 categorical variables. Therefore, *p* = 0.0042 was used as the significance level. Our results showed that all the transformed continuous independent variables in the model (ln_WBC, ln_Alb, ln_Blood glucose, ln_SMA and ln_VFA) have a *p*-value > 0.0042, indicating that each of them had a linear relationship with the outcome variable (Supplementary Table S1).

#### Multi-collinearity test

The Variance Inflation Indicator (VIF) and tolerance factor are used to show whether a predictor has a strong linear relationship with the other predictor(s). If the tolerance is <0.1 or the VIF is >10, then there is multi-collinearity. In this case, the variance inflation factor (VIF) for all the independent variables was between 1.024 and 1.109, while tolerance was between 0.902 and 0.977, so there is no multi-collinearity (Supplementary Table S2).

#### Influential data points test

Cook's distance is used in regression analysis to identify influential data points that may negatively affect your regression model. Any point with a Cook's distance over 4/n (where n is the total number of data points) is considered to be an outlier. Our results showed that the Cook's distance for each observation is less than the threshold, indicating that there are no influential data points in the dataset (Supplementary Figure S1).

**Figure 2:**
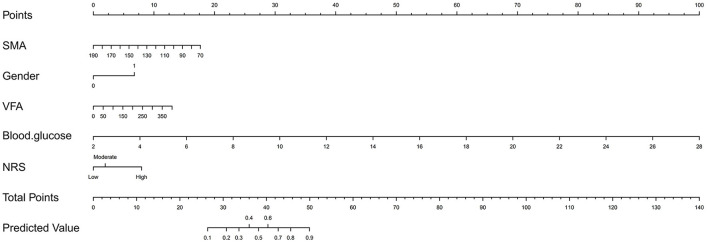
Nomogram for predicting postoperative anastomotic leakage in patients with colorectal cancer.

Variables with *p* < 0.05 were further included in the multivariable model. The results showed that gender, blood glucose, NRS, SMA and VFA were independent risk factors for the occurrence of AL (Table 3). Regarding gender, men had a higher risk of developing AL than women (OR 3.746, 95% CI, 1.503–9.335, *p* = 0.005). Further, blood glucose proved to be a significant independent predictor, AL was more likely to occur in patients with higher blood glucose (OR 2.011, 95% CI, 1.444–2.802, *p* = 0.000). Additionally, lower SMA and higher VFA were more likely to develop AL (OR 0.974, 95% CI, 0.958–0.990, *p* = 0.001; OR 1.006, 95% CI, 1.001–1.012, *p* = 0.027). Concerning NRS, the risk of AL was the highest in NRS ≥5 (OR 4.735, 95% CI, 1.068–20.988, *p* = 0.041). On this basis, we established a nomogram (Figure 2). In the AL nomogram, blood glucose accounted for the largest proportion, followed by SMA, VFA, NRS and gender.

**Table 3:** Multivariate analysis of prognostic factors for AL.

	**Std. Err**	**Exp (B)**	**95% CI**,	***p***
**Gender**	0.466	3.746	(1.503~9.335)	0.005^*^
**WBC, 10** ^**9**^ **/L**	0.086	1.140	(0.963~1.350)	0.128
**Alb, g/L**	0.038	0.963	(0.894~1.036)	0.311
**Blood glucose, mmol/L**	0.169	2.011	(1.444~2.802)	0.000^*^
**NRS**				
<3	/	/	/	0.117
≥3 and <5	0.481	1.378	(0.537~3.534)	0.505
≥5	0.760	4.735	(1.068~20.988)	0.041^*^
**SMA, cm** ^**2**^	0.008	0.974	(0.958~0.990)	0.001^*^
**VFA, cm** ^**2**^	0.003	1.006	(1.001~1.012)	0.027^*^

WBC, white blood cell; Alb, albumin; NRS, nutrition risk screening; SMA, skeletal muscle area; VFA, visceral fat area.

* p < 0.05.

### Predictive model performance

**Figure 3:**
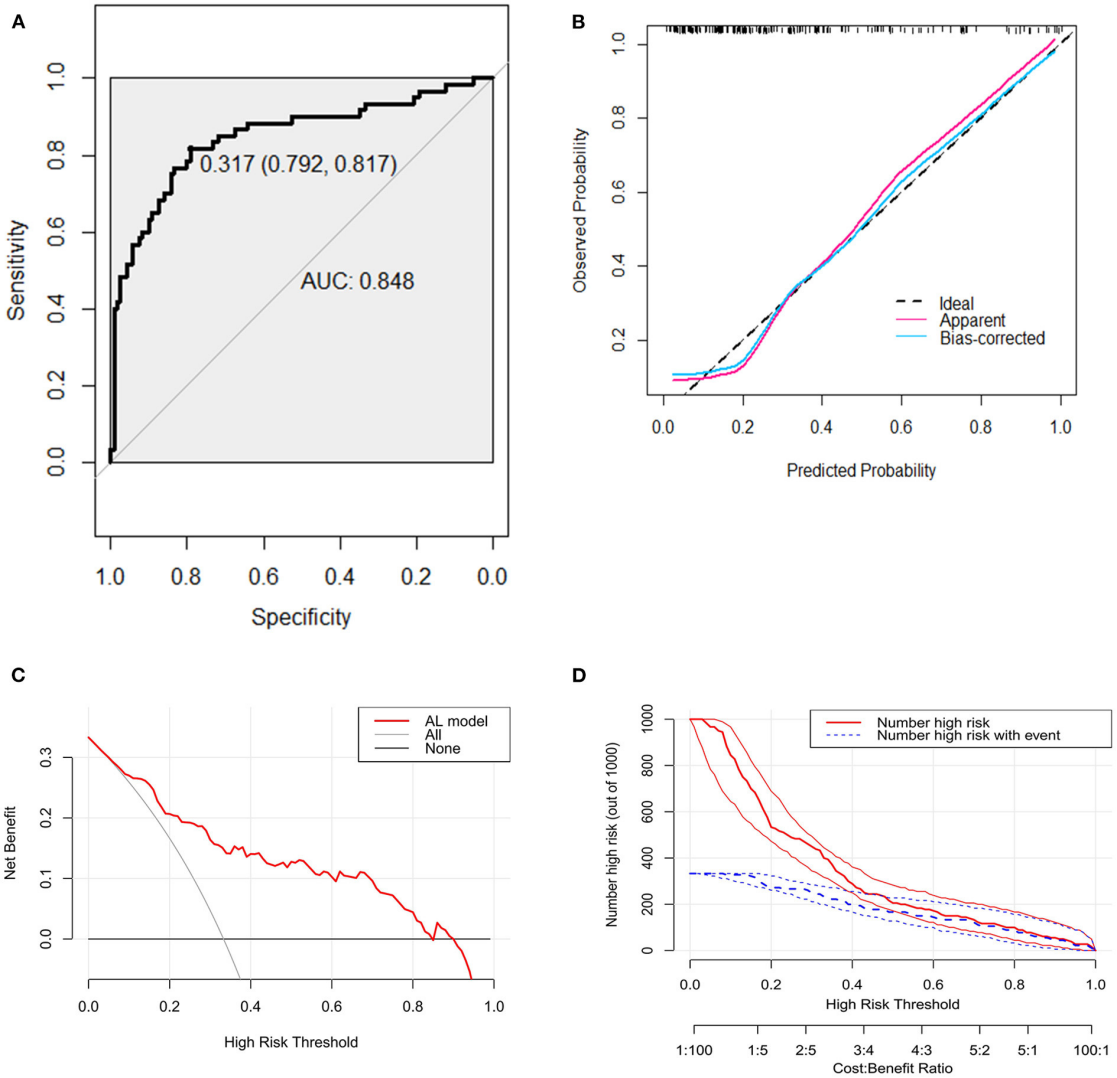
Receiver operating characteristic curve, calibration curve, decision curve analysis (DCA), and clinical impact curve for predicting anastomotic leakage (AL) in patients with colorectal cancer (CRC). **(A)** Area under the curve for predicting the AL of patients with CRC. **(B)** Calibration plot of the prediction model (bootstrap method, 1,000 repetitions). **(C)** Decision curve analysis of the model for predicting the risk of AL for patients with CRC. The x-axis means the threshold probability and the y-axis means the net benefit. The black line assumes that no patient has AL. The gray line assumes that all patient has AL. **(D)** Clinical impact curve. The red curve (Number high risk) represents the number of people classified as positive (high risk) by the model at each threshold probability; the blue curve (Number high risk with event) is the number of true positives under each threshold probability.

The bootstrap procedure was employed for internal validation and tested the performance of predictive model with 1,000 repetitions. Based on the receiver operating characteristic (ROC) analysis, the area under the curve (AUC) of the nomogram was 0.848 (95% CI, 0.781–0.914), indicating that the nomogram can predict AL effectively (Figure 3A). Furthermore, the calibration plot of the nomogram demonstrated that the observed and predicted probabilities of AL correlated well in our model (Figure 3B). The Hosmer-Lemeshow goodness-of-fit test of the nomogram harvested a non-significant statistic in the cohort with P-values as 0.343. Then we performed decision curve analysis (DCA) to evaluate the net benefit for patients in clinical practice. The results suggested that the nomogram has good clinical application value (Figure 3C). The clinical impact curve showed good consistency between the prediction of the nomogram and the actual observed outcomes (Figure 3D).

### Correlation analysis of BMI to CT body composition

The results of the correlation analysis of the BMI with CT body composition were shown in Supplementary Figures S2–S5. BMI was significantly positively correlated with VFA (*R* = 0.57, *P* < 0.001), SMA (*R* = 0.29, *P* < 0.001), SAT (*R* = 0.54, *P* < 0.001), but not with IMF (*R* = 0.13, *P* = 0.082).

## Discussion

AL is one of the most serious complications after CRC surgery, resulting in severe morbidity, prolonged hospital stays, intensive use of medical resources, and increased risk of death. Previous studies have shown that obesity, gender, history of diabetes, etc. are risk factors for the occurrence of AL (17). However, there are few studies on the clinical value of body composition determined by preoperative CT images in predicting postoperative AL.

In our study, gender, blood glucose, NRS, SMA and VFA were independent factors for AL occurrence in CRC. Blood glucose is the most significant influencing factor, and the risk of AL was significantly higher in patients with higher preoperative blood glucose levels. Similar to our findings, the study by Kotagal et al. showed that diabetic (DM) patients had significantly higher rates of postoperative adverse events than non-diabetic (NDM) patients. Even among NDM patients, hyperglycemic patients had an increased risk of adverse events compared with normoglycemic patients. A dose-response relationship exists between blood glucose levels and composite adverse events in NDM patients (18). The underlying mechanism may be related to the inflammatory response. Hyperglycemia exacerbates inflammatory, oxidative stress responses and cytokine, potentially creating a vicious circle (19–21). Resolution of hyperglycemia was associated with normalization of the inflammatory response (20). Our study revealed that SMA and VFA are two other important risk factors. Patients with lower SMA or higher VFA are more likely to develop AL. A retrospective study of 2011 patients showed that VFA was an independent risk factor for AL in the elective colon resection group. A study by Wang et al. including 859 patients also reported similar results that preoperative high VFA was an independent risk factor for postoperative complications. Nowadays, adipose tissue is considered an endocrine and paracrine organ whose function affects many physiological processes, including inflammation, fat and glucose metabolism, energy balance. The levels of VEGF and IL-6, along with the proportion of CD8+ T cells and NKT cells, were significantly increased in visceral adipose tissue (22). Inflammatory cells, including macrophages and T cells, are abundant in visceral adipose tissue, and excess visceral adipose tissue induces a chronic systemic inflammatory state with associated insulin resistance and metabolic dysfunction (23). These may be potential causes of postoperative AL. Due to the influence of gastrointestinal tumors, patients may experience gastrointestinal symptoms such as loss of appetite and reduced dietary intake before surgery. In addition, general malaise and mental anxiety also reduce nutritional intake and physical activity, resulting in reduced skeletal muscle mass. Some studies show that nutritional support can increase calorie and protein intake in patients (24, 25). Decreased skeletal muscle wasting favors maintenance of the amount of myokines secreted by skeletal muscle and is expected to improve tolerance to surgery. A study by Shiro Fujihata et al. showed that lower skeletal muscle mass index, especially in the erector spinae muscle, was significantly associated with higher AL (26). Therefore, adequate nutritional support before surgery is necessary to prevent the occurrence of AL. In addition, male is also an independent risk factor for postoperative AL, which is similar to previous studies (27–29). The content of androgens in intestinal microcirculation may be related to anastomotic healing (30). An animal experiment found that in the early stage of wound healing, the collagen metabolism in the colonic anastomosis of male rats was worse than that of female rats (31). The NRS is a nutritional risk screening tool that reflects the patient's current nutritional status. Several studies have reported that NRS nutritional assessment results are associated with outcomes of in-hospital patients (32–34). Patients with nutritional risks who require colorectal cancer surgery should be carefully managed.

The results of correlation analysis between BMI and body composition showed that BMI was significantly positively correlated with VFA, SMA and SAT, but not with IMF. In multivariate analysis, VFA and SMA were independent risk factors for postoperative AL, suggesting that VFA and SMA may be more sensitive predictors of AL than BMI (35, 36).

Nomogram is a graphical representation of a complex mathematical formula (37), and one of its main advantages is the ability to estimate individualized risk based on patient and disease characteristics. The nomogram can incorporate disease-related continuous and categorical variables into the model, with a friendly interface, which is helpful for clinical decision-making and promotes the development of personalized medicine.

Commonly used nutritional status assessment tools, such as BMI and NRS scores, can only observe the overall status, but cannot obtain individual components of the body, such as regional fat distribution and muscle composition. More and more studies have confirmed that nutritional assessment based on body composition can better reflect the patient's metabolic status and physiological reserve, and may be a decisive factor affecting postoperative outcomes (38). Almost all patients with CRC underwent whole abdominal CT before surgery, so CT images of L3 level are very easy to obtain. At the same time, with the help of nomogram, clinicians can predict the occurrence of AL in patients with more basis before surgery. For those patients with high predicted probability and preoperative nutritional risk, active nutritional support should be provided, which will help maintain proper nutritional status and reduce the number and severity of postoperative complications (39). In addition, the predicted probability can also provide a certain reference when clinicians are hesitant to perform preventive ostomy.

There are some limitations to our study. First, this is a single-center retrospective study with a small sample size, the established nomogram lacks external validation, and its performance in an external cohort remains to be studied. Second, although we selected controls as representative as possible, selection bias may still exist. Third, we only assessed preoperative body composition and lacked information on postoperative body composition changes and prognostic data. Further, some indicators were meaningful in univariate analysis but not in multivariate analysis, which may be due to the small sample size. Therefore, larger prospective multicenter studies are necessary in order to approve these preliminary results.

## Conclusion

In conclusion, based on the clinical data and imaging information we collected, we developed a nomogram that can predict AL in patients with CRC. The performance of nomogram was verified by various methods, and the results showed that the nomogram had high accuracy and reliability in predicting AL. Preoperative prediction of AL can help surgeons make appropriate therapeutic decisions in clinical practice.

## Data availability statement

The original contributions presented in the study are included in the article/Supplementary material, further inquiries can be directed to the corresponding authors.

## Author contributions

S-LL and YL: conceptualization and supervision. SX, S-LL, T-YW, and Z-TY: data curation, methodology, and software. SX and Y-KY: writing original draft. S-LL: writing—review and editing. All authors contributed to the article and approved the submitted version.

## Funding

The study was supported by the National Natural Science Foundation of China (Grant No. 81802888), the Key Technology Research and Development Program of Shandong (No. 2018GSF118088), and the General Financial Grant from the China Postdoctoral Science Foundation (No. 2016M592143).

## Conflict of interest

The authors declare that the research was conducted in the absence of any commercial or financial relationships that could be construed as a potential conflict of interest.

## Publisher's note

All claims expressed in this article are solely those of the authors and do not necessarily represent those of their affiliated organizations, or those of the publisher, the editors and the reviewers. Any product that may be evaluated in this article, or claim that may be made by its manufacturer, is not guaranteed or endorsed by the publisher.
