# Supplementary material for: Development and validation of a nomogram to predict anastomotic leakage in colorectal cancer based on CT body composition
Source: Front Nutr. 2022 Sep 7;9:974903. doi: 10.3389/fnut.2022.974903 (PMC9490075; doi:10.3389/fnut.2022.974903)
Supplement: Supplementary file 1 [file Data_Sheet_1.docx]

Supplementary Material

Supplementary Figure 1. Cook’s distance is used in the analysis of outliers. In this study, the cook’s distances of the 18th, 22th and 120th samples are large, but they are all less than 1. Therefore, we do not consider these samples as outliers.


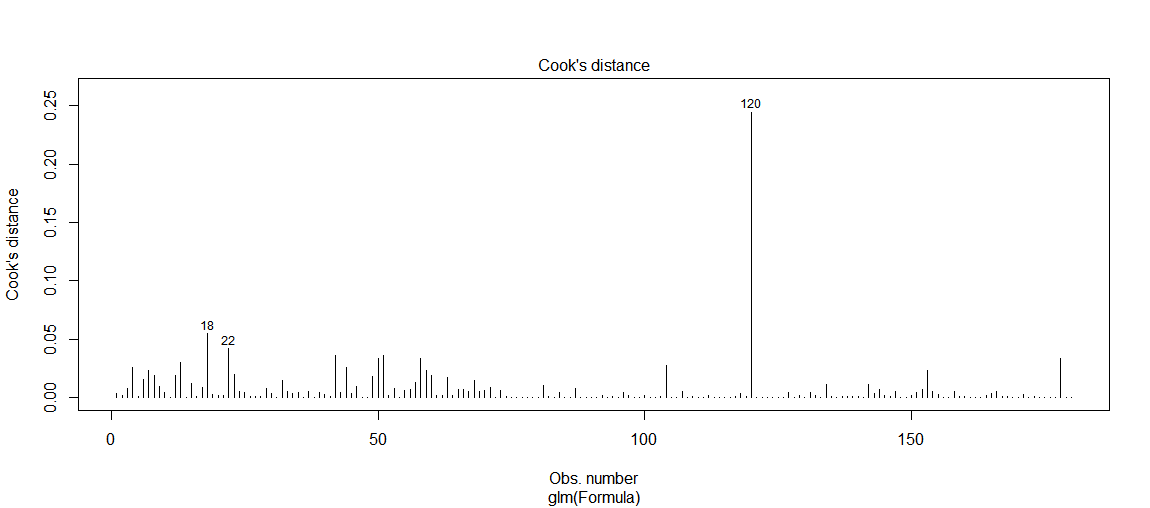


Supplementary Figure 2. Spearman’s correlation between visceral fat area (VFA) and body mass index (BMI).


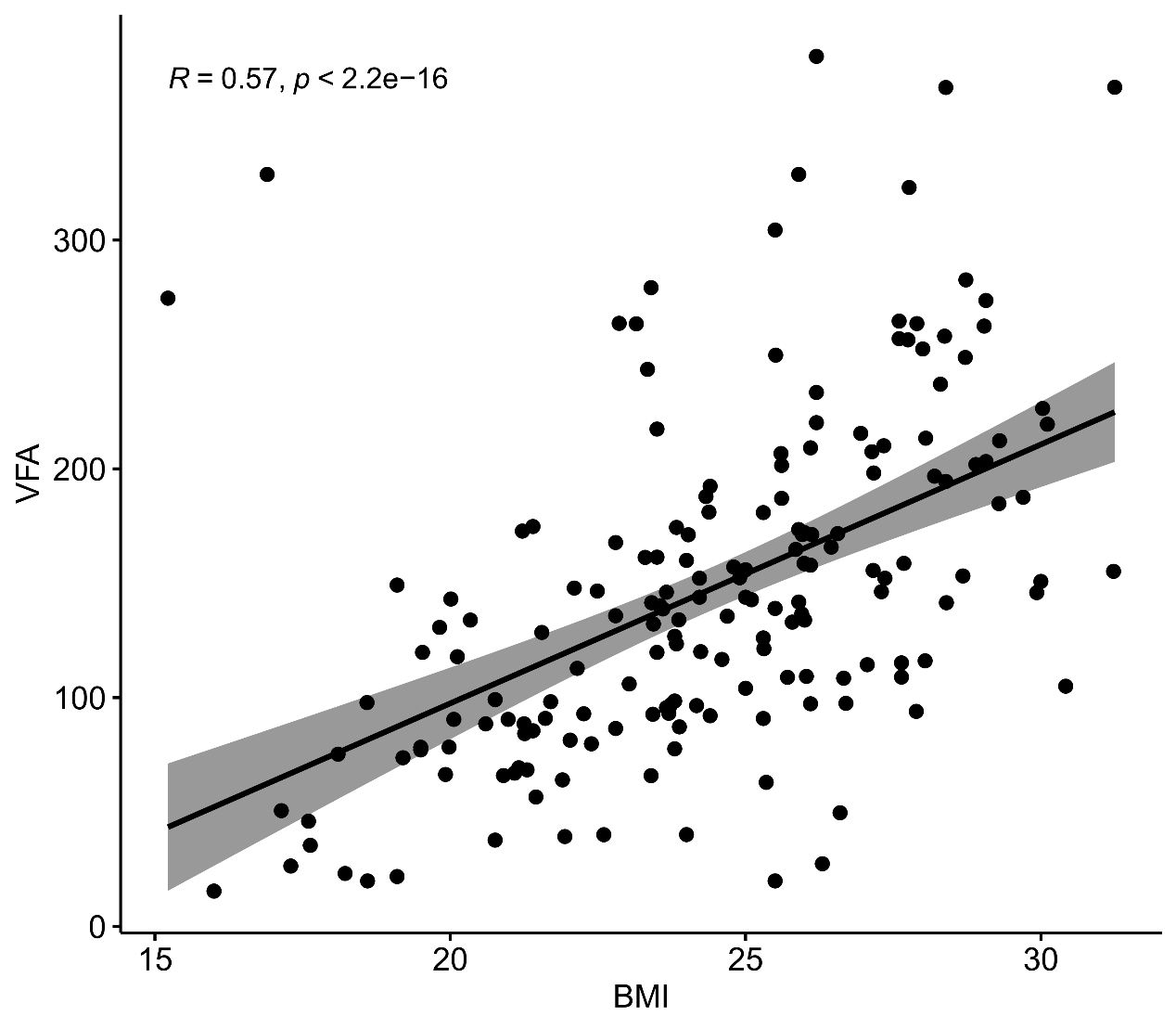


Supplementary Figure 3. Spearman’s correlation between skeletal muscle area (SMA) and body mass index (BMI).


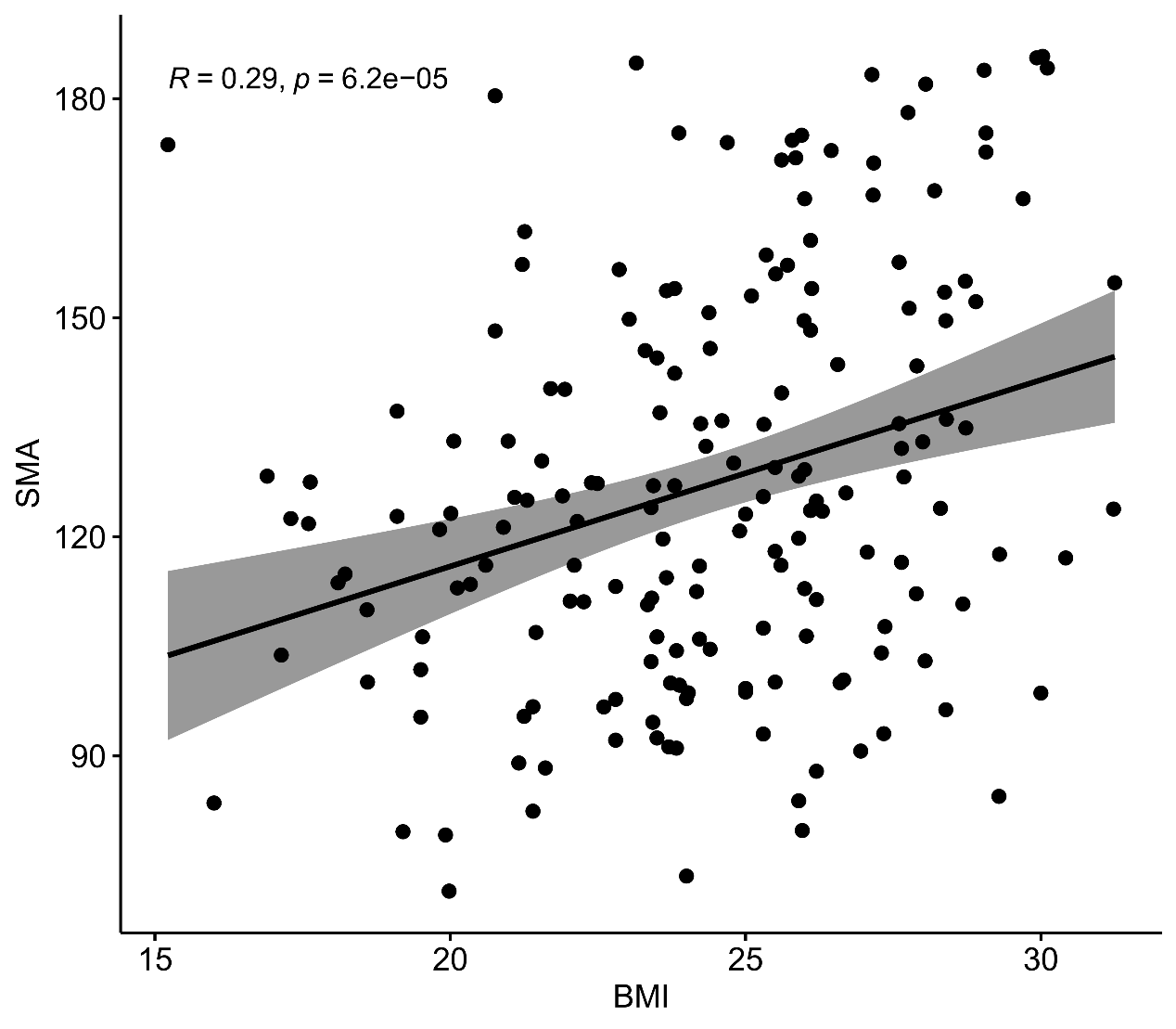


Supplementary Figure 4. Spearman’s correlation between subcutaneous fat area (SFA) and body mass index (BMI).


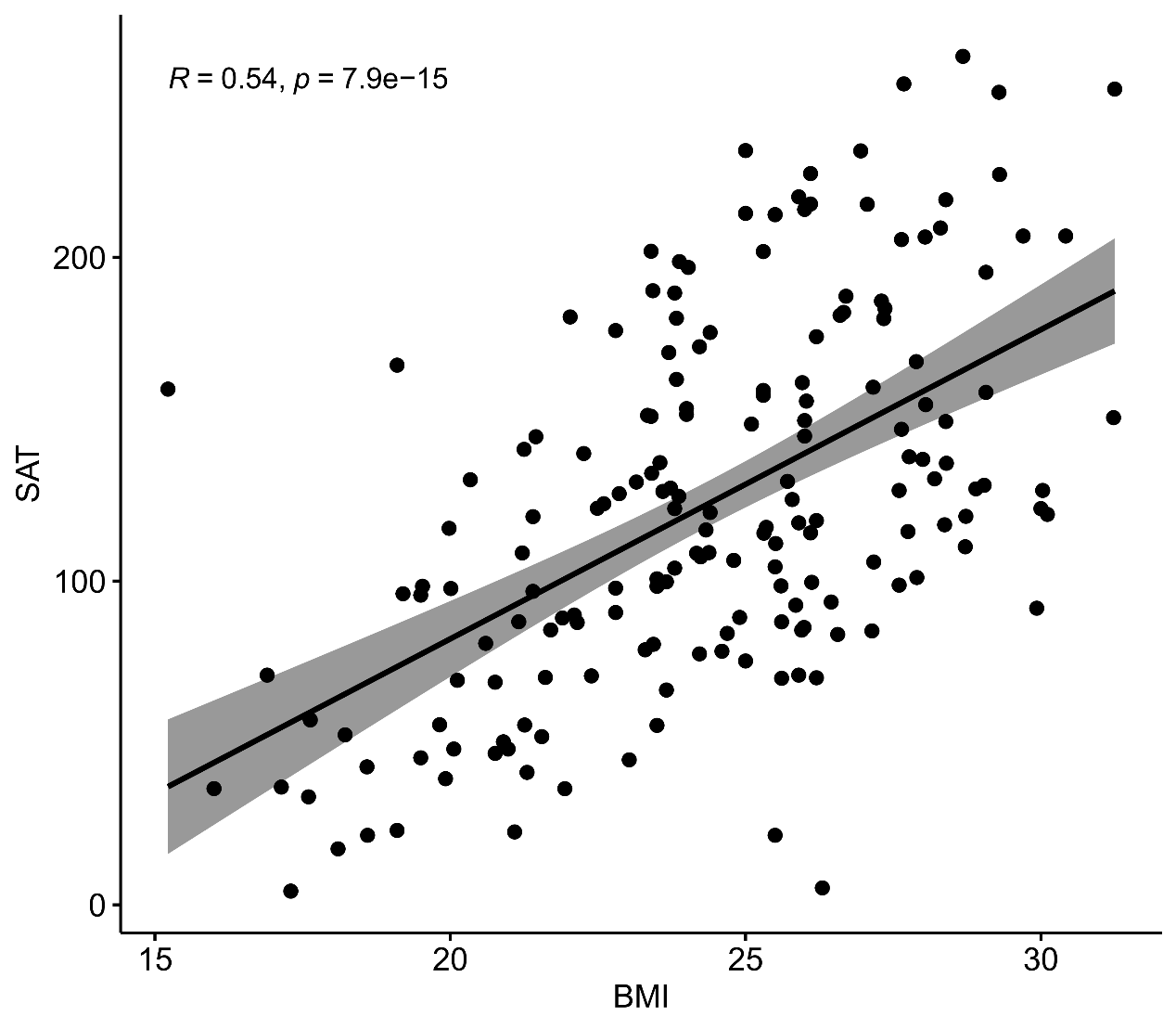


Supplementary Figure 5. Spearman’s correlation between intermuscle fat area (IMFA) and body mass index (BMI).


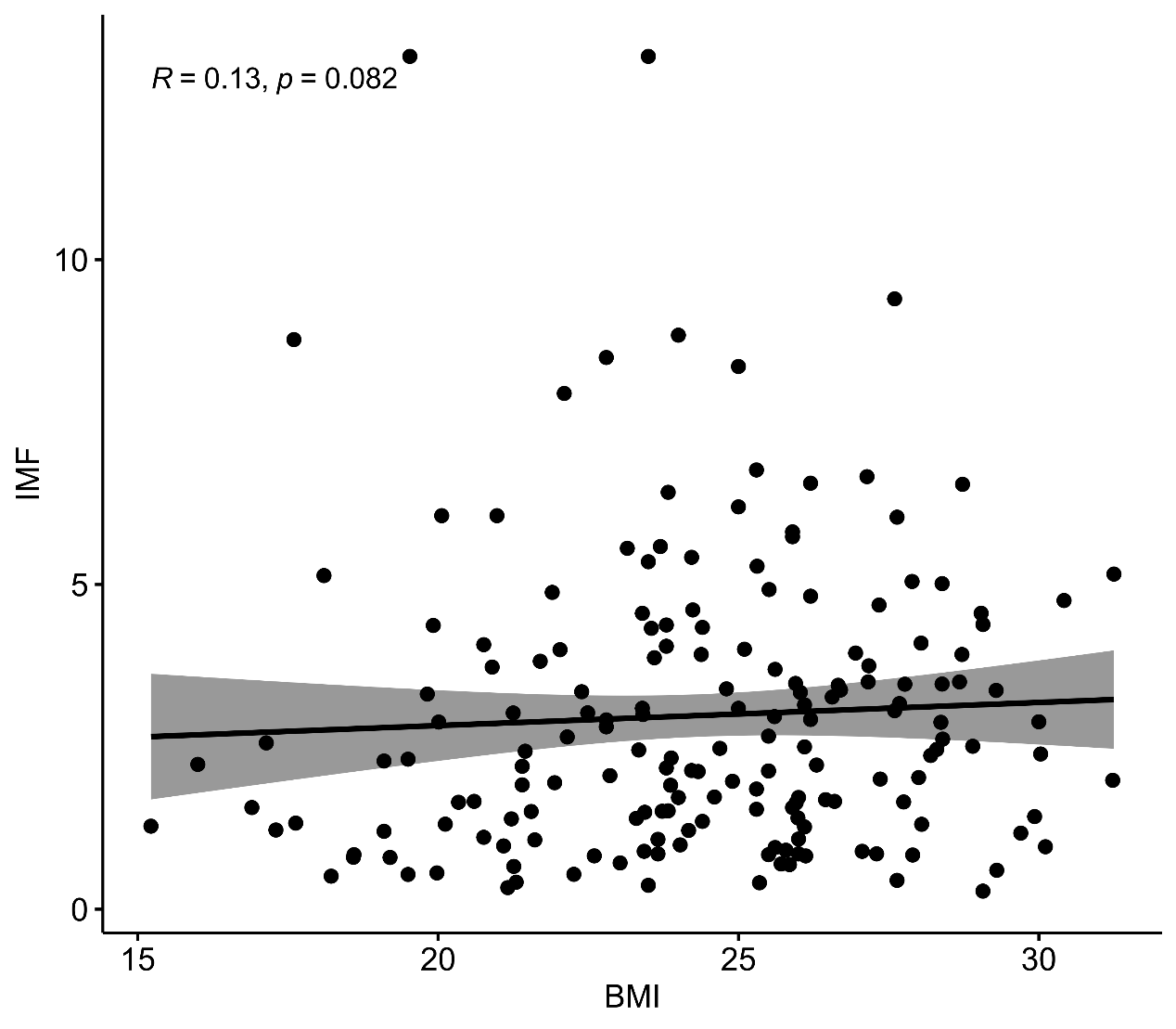


Supplementary Table 1. Variables in the Equation. Box-tidwell method tests the linear relationship between continuous independent variables and logit conversion values of dependent variables.

|  | **S.E.** | **Sig.** | **Exp(B)** |
| --- | --- | --- | --- |
| **WBC by ln_WBC** | 0.339 | 0.739 | 0.893 |
| **Alb by ln_Alb** | 0.412 | 0.236 | 0.614 |
| **Blood glucose by  ln_Blood glucose** | 0.364 | 0.072 | 0.520 |
| **SMA by ln_SMA** | 0.065 | 0.765 | 0.981 |
| **VFA by ln_VFA** | 0.010 | 0.010 | 0.975 |
| **Constant** | 19.014 | 0.115 | 0.000 |

Supplementary Table 2. Collinearity Statistics. The variance inflation factors (VIF) are all less than 10, indicating that there is no multicollinearity between independent variables. Dependent Variable: AL

|  | **Tolerance** | **VIF** |
| --- | --- | --- |
| **SMA** | 0.949 | 1.053 |
| **VFA** | 0.945 | 1.058 |
| **Blood glucose** | 0.939 | 1.065 |
| **Alb** | 0.965 | 1.036 |
| **WBC** | 0.958 | 1.043 |
| **Gender** | 0.902 | 1.109 |
| **NRS** | 0.977 | 1.024 |
